# Supplementary material for: Integrated Analysis of the Lung Microbiome and Metabolome Reveals Associations Between Amino Acid Metabolism and Pulmonary Fibrosis in a Bleomycin-Induced Mouse Model
Source: Int J Mol Sci. 2026 Jun 30;27(13):5895. doi: 10.3390/ijms27135895 (PMC13362081; doi:10.3390/ijms27135895)
Supplement: Supplementary file 1 [file ijms-27-05895-s001.zip › result/6.MetGSEA/6-MetGSEA-readme.pdf]

MetGSEA Readme

|-- 6. MetGSEA **【GSEA 富集分析】**
| -- *.vs.* 【比较的样品对目录】
| |-- {all} 【不同离子模式目录】
| | |-- *.vs.*_{all}_KEGG_gsea_result.{xls,xlsx} 【 GSEA 富集结果】
| | |--enplot_MAP*.png 【 GSEA 富集结果展示图】
| | |--MAP*_. {xls,xlsx} 【GSEA 富集条目详细结果（默认取 NSE 值最大 20 个和最小 20 个条目）】

***.vs.*_{all}_KEGG_gsea_result.{xls,xlsx}**

第一列： MapID, 富集的 KEGG PATHWAY 的 ID;
第二列： KEGG_Term, 富集的 KEGG PATHWAY 名称;
第三列： Pvalue , GSEA 富集分析的 Pvalue;
第四列： AdjustedPv, 校正后的 Pvalue;
第五列： Size，功能条目包含该比较对鉴定代谢物的数量;
第六列： ES，功能条目 GSEA 富集得分 ;
第七列： NES， 矫正后 GSEA 富集得分;

MAP*_. {xls,xlsx}

第一列： Compound_ID ，代谢物 ID;
第二列： Name，代谢物的名称（数据库中代谢物的名称） ;
第三列： RANK IN LIST，代谢物在该比较对排序中的位置;
第四列： RANK METRIC SCORE ，代谢物在该比较对中的排序评分;
第五列： RUNNING ES，累计的 Enrichment score;
第六列： CORE ENRICHMENT，Yes 表示是属于核心代谢物，No 则不属于（核心代谢物：对 ES 的贡献最大的代谢物）;
